# Improving PHIRI performance and scalability: working within EGI-ACE

**DOI:** 10.1093/eurpub/ckac129.470

**Published:** 2022-10-25

**Authors:** G Sipos

**Affiliations:** EGI, Amsterdam, Netherlands

## Abstract

EGI-ACE is a 30-month H2020 project (Jan 2021 - June 2023) with a mission to empower researchers from all disciplines to collaborate in data- and compute-intensive Open Science, enabled by free-at-point-of-use services that are delivered through the European Open Science Cloud (EOSC). EGI-ACE delivers the EOSC Compute Platform (ECP), a federated system of compute and storage infrastructure extended with platform services to support diverse types of data processing and data analytics cases. The ECP currently includes High Throughput Compute (HTC) and Cloud Compute facilities, and will broaden its scope with High Performance Compute services later in 2022. The platform layer of the ECP provides assistance for single sign-on, transfer and federation of distributed data, interactive computing, management of large numbers of jobs, orchestration of compute clusters, AI and machine learning tasks. There are over 25 thematic services in EOSC that build on the ECP, and deliver scalable data analysis for different domains, from astrophysics, through life sciences, environmental sciences, to humanities. PHIRI participates in EGI-ACE as one of the ‘Early Adopters’ of the ECP. Under the EGI-ACE workplan PHIRI will explore reproducible population health workflows with the use of cloud computing, single-sign-on, Jupyter Notebooks and Binder services of the ECP. The tests will enable PHIRI to scale out existing data analysis notebooks to big capacity machines, to reproduce simulations and models across users, and to overall validate the technological and sustainability approaches of EGI-ACE. PHIRI will also advise the project on best ways to introduce secure processing capabilities within the ECP services.

